# Plant-Based Phenolic Molecules as Natural Preservatives in Comminuted Meats: A Review

**DOI:** 10.3390/antiox10020263

**Published:** 2021-02-09

**Authors:** Michel M. Beya, Michael E. Netzel, Yasmina Sultanbawa, Heather Smyth, Louwrens C. Hoffman

**Affiliations:** 1 Centre for Nutrition and Food Sciences, Queensland Alliance for Agriculture and Food Innovation (QAAFI), The University of Queensland, Brisbane, QLD 4072, Australia; michel.beya@uqconnect.edu.au (M.M.B.); m.netzel@uq.edu.au (M.E.N.); y.sultanbawa@uq.edu.au (Y.S.); h.smyth@uq.edu.au (H.S.); 2 Department of Animal Sciences, Stellenbosch University, Private Bag X1, Matieland, Stellenbosch 7602, South Africa

**Keywords:** plant extracts, polyphenols, comminuted meat, natural preservatives, antimicrobial, antioxidant

## Abstract

Comminuted meat products are highly susceptible to safety and quality degradation partly because of their large interfacial area in the emulsion. The food industry extensively uses synthetic chemical preservatives to delay that degradation which is caused by microbial growth, enzyme activities and oxidation reactions. However, due to the potential health damage (e.g., cardiovascular diseases, neurodegenerative diseases, cancers among others) synthetic preservatives in meat may cause, consumers are becoming skeptical to buy meat products containing such additives. In the meat industry, the interest of finding natural food preservatives is intensifying. Polyphenolic-rich plants used as natural food preservatives offer the best alternative for a partial or a complete replacement of their synthetic counterparts. They can be extracted from natural sources such as olives, fruits, grapes, vegetables, spices, herbs, and algae, and among others. The common feature of these phenolic compounds is that they have one or more aromatic rings with one or more -OH group which are essential for their antimicrobial and antioxidant properties. This review article is intended to provide an overview of the plant-based phenolic molecules used as natural food preservative, their antimicrobial and antioxidant mechanism of action, and their potential application in comminuted meat.

## 1. Introduction

A report from the Food and Agriculture Organization (FAO) [1] predicted that the degradation of the world ecosystem in addition to the projected global population growth will challenge humanity‘s ability to feed itself. Moreover, each year, the loss of globally produced food is estimated at 40% with microbial food spoilage and quality degradations being some of the major causes thereof [2]. On the other hand, the global average per capita consumption of meat is on the rise driven by increasing average per capita incomes and population growth [3]. Consequently, this place pressure on the global meat supply chain and government institutions to find sustainable solutions to address food security. Meat, particularly comminuted meat, is a good source of proteins, trace elements such as iron and zinc, vitamin B12, and essential amino acids, all critical components for a balanced and healthy human diet. However, partly due to the large interfacial area in the emulsion, comminuted meat is highly susceptible to oxidative degradation and microbial spoilage [4].

Since the industrial revolution, the food industry has been using, with relative success, synthetic chemical preservatives such as sodium metabisulfite [5], potassium sorbate [6], sodium benzoate [7], butylated hydroxytoluene (BHT) [8], butylated hydroxianisole (BHA) [9], sodium nitrite [10] and more to ensure the safety and quality of meat products by inhibiting and preventing the growth of spoilage and pathogenic cells and delaying the oxidation of lipids and proteins. Because of the growing body of evidence about the potential toxicity of these preservatives, consumers have become skeptical and are asking for a healthier food [11]. In Australia, a national survey reported that 45% of Australians were concerned about the safety and quality of foods they buy due to potential hazards of synthetic chemical preservatives in foods [12]. Plant-based preservatives are generally regarded as safe (GRAS) without any documented detrimental impact and have been reported to contain bioactive compounds that play an important role in the preservation of foods.

Several bioactive compounds naturally occurring in plants have antioxidant and antimicrobial properties and may play crucial roles in the preservation of comminuted meat products. Plant-based bioactive compounds are generally classified as phenolic compounds found in a variety of plants, fruits, herbs, and spices [13]. Numerous studies on plant polyphenols, as natural preservatives in comminuted meat, has been reported. These include rosemary [14], clove extracts [15], green tea [16], turmeric [17], and *Moringa oleifera* leaf [18]. In their review paper, Das and co-authors [19] noted that although there are many types of bioactive molecules of natural origin for meat preservation, such as those from animal and microorganisms, the greatest interest for meat manufacturing is centered on using plant extracts rich in bioactive molecules.

Polyphenols are recognized for their powerful antimicrobial and antioxidant properties. Their bioactivity is based on their capability to: scavenge free radicals and reactive oxygen/nitrogen species (ROS/N), reduce oxidized intermediates, induce metal chelation, and inhibit enzymes responsible for the formation of free radicals [20]. These molecules differ in terms of their physiochemical structure and molecular weight; and they consist of at least one aromatic ring, to which at least two hydroxyl groups are attached [21]. The bioactivity of polyphenols is dependent on the configuration of the molecules, and the position and number of the hydroxyl group(s) in that molecule [22]. Polyphenolic compounds, based on their structure, are divided into four major groups: phenolic acids, flavonoids, stilbenes and lignans [21].

Reflecting on the significant number of recently published journal articles focusing on the antioxidant and antimicrobial properties of polyphenols as natural preservatives in comminuted meat, the aim of this review is to highlight and summarize the relevant findings of selected plants. The scope of this review is related to bioactivity of polyphenolic-rich plant materials, their antimicrobial and antioxidant mechanism of actions, their application in comminuted meats. Challenges in the in-situ applications of plant derived bioactive compounds and government regulations will also be highlighted.

## 2. Plant-Based Bioactive Molecules for Food Preservation

**Figure 1:**
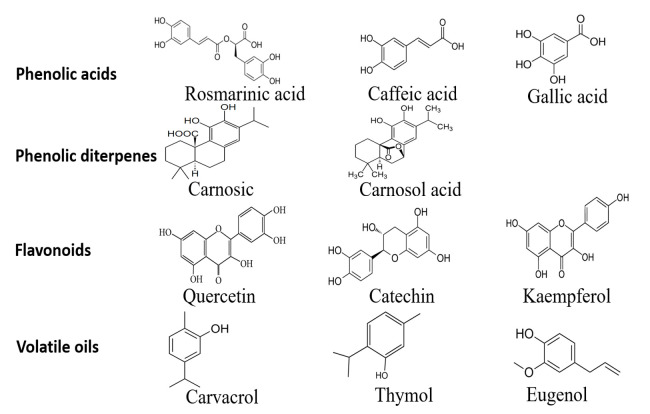
Chemical structures of selected plant-based phenolic compounds.

Many plants have been reported to contain some antimicrobial and antioxidant properties due to the presence of bioactive compounds. This bioactivity is mainly attributed to a variety of phenolic molecules (as shown in Figure 1) which are fundamentally related but vary in their conformations and quantity depending on the specific source [23]. In the plant extracts, phenolic compounds consist of phenolic acids (e.g., rosmarinic acids, caffeic acids and gallic acid), phenolic diterpenes (e.g., carnosol and carnosic acid), flavonoids (e.g., quercetin, catechin, apigenin, naringenin, kaempferol and hesperetin), volatile oils (e.g., carvacrol, thymol, eugenol, and menthol) [24] and, many other bioactive compounds have been isolated. The common characteristic of these phenolic compounds is their ability to inhibit or prevent the growth of spoilage and pathogenic microorganisms and to slow down the oxidative reactions within the food system.

## 3. Extraction of Plant Bioactive Compounds for Food Application

The extraction is an important step to isolate polyphenol molecules from plant materials. The concentration of these bioactive molecules in the extract can differ considerably depending on the type of plant and the technology applied. Methods of extraction include the use of solvents, distillation, pressing and sublimation [25]; and among which, the solvent extraction is the most used method. There are several techniques used, both in industry and in research, for the extraction and recovery of bioactive compounds from plant sources. These include techniques such as Soxhlet, maceration and stirring [26], ultrasound assisted extraction [27], pressurized liquid extraction (PLE) [28], subcritical water extraction [29], supercritical fluid extraction (SFE) [30], microwave assisted extraction, pulse electric field extraction and enzyme-assisted extraction [31]. The selection of a particular extraction method and technique is conditional on the physico-chemical nature of compounds to be extracted, whilst the cost and safety of the method should also be considered.

The extraction processes of bioactive molecules from plant materials consists of the following phases: the solvent penetrates and diffuses into the solid matrix; (2) the solute dissolves into the solvent; (3) the solute is diffused out of the solid matrix; the extracted solutes are collected [25]. The chemical nature of the extraction solvent, particle size of the plant materials, the ratio solvent-to-solid, the extraction temperature and the extraction duration influence the efficiency of the extraction. The choice of the extraction solvent depends largely on the desired fraction to be extracted; e.g., sesame oil was better extracted with less polar solvent [32]. However, membrane-associated lipids are more polar and required the use of a polar solvent capable of breaking the hydrogen bonds or the electrostatic forces.

The storage conditions of the plant extract have implications on the shelf life and the bioactivity strength of the extract. Research findings from Birch leaf hydrolysable tannins suggested freeze-and vacuum-drying at −20 °C provided a more reliable result and longer shelf life whereas storage at 4 °C and room temperature decreased the concentration of the pedunculagin derivatives over a period of one year [33]. These results can be explained by the fact that removing oxygen from the package of Birch leaves and cooler temperature during storage slowed down the oxidation processed and eventually prevented microbial growth that might impacted on the bioactivity potential of the plant extracts. In agreement with these findings, the prolonged storage of blueberry extract at room temperature significantly decreased the phenolic compounds and their biological activities [34]. An effective extraction of plant bioactive molecules ought to consider all factors to ensure the integrity of the extracts.

## 4. Antioxidant Mechanism of Action

**Figure 2:**
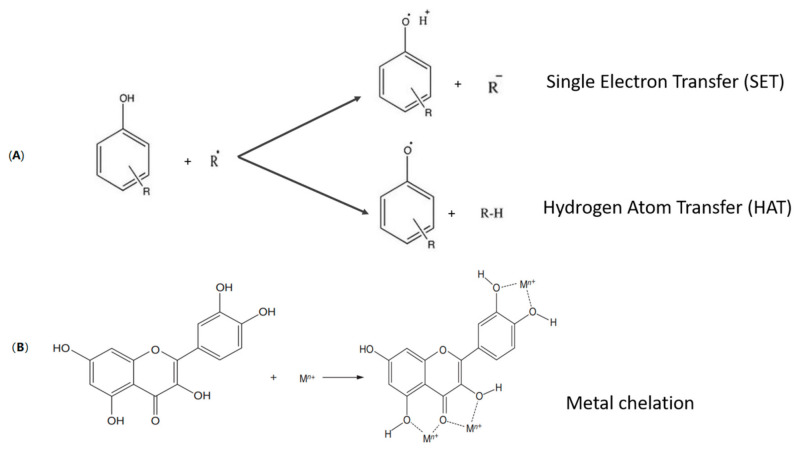
Schematic representation of an antioxidant’s mechanism of actions. Single electron transfer (SET) and hydrogen atom transfer (HAT). (**A**) SET involves the ionization potential of the antioxidant by transferring so as to deactivate a free radical. HAT involves the bond dissociation enthalpy in which the free radical removes one hydrogen atom from the antioxidant. (**B**) Metal chelation involves the binding of a metal (oxidant) to form a complex ring-like structure.

Fundamentally, the antioxidant mechanism involves a concerted transfer of the H-atom from a phenolic molecule to the radical thereby inhibiting either the early free radical formation or, stopping the generation of additional free radicals which may propagate the oxidation reaction further [11]. There are two main category of antioxidants based on their mechanism of actions [35]: (1) primary antioxidants that disrupt the oxidative free radical’s chain reaction by donating electrons or hydrogen atoms from the hydroxyl group to stabilize the free radicals, and as a result, inhibit or slow down the initiation phase and disturb the propagation stage of autoxidation; (2) secondary antioxidants that deactivate the singlet oxygen, chelate metal ions, absorb UV radiation, scavenge oxygen and help to regenerate the primary antioxidant as schematized in Figure 2.

The bioactivity of antioxidants is mainly due to the combination of aromatic rings and OH-groups that (re-)assemble their chemical structure to be able to bind and prevent or, slow down lipid and protein oxidations [36]. This bioactivity of a plant-based polyphenol increases with the number of OH-groups in its molecular structure. For example, among flavones and flavanols, the scavenging of peroxyl and hydroxyl increased respectively according to the number of OH-groups [37]. In addition, the configuration of the aromatic ring in the antioxidant molecular structure influences its activity. For instance, the scavenging ability of luteolin substantially exceeded kaempferol’s; this is because kaempferol lacks the B-ring catechol in its configuration [38].

Furthermore, transition metals have the capacity to induce the oxidation of lipids by decomposing lipid hydroperoxides to generate lipid alkyl radicals. Polyphenols can prevent lipid peroxidation by binding and neutralizing free radicals [35]. However, this depends on the configuration of the molecules, and the number and the position of the OH-group [39]. For example, Kelley and co-workers [36] demonstrated that both the configuration and total number of OH-groups on the polyphenol molecule significantly influence several mechanisms of antioxidant activities.

## 5. Antimicrobial Mechanism of Action for Preservation in Food Systems

**Figure 3:**
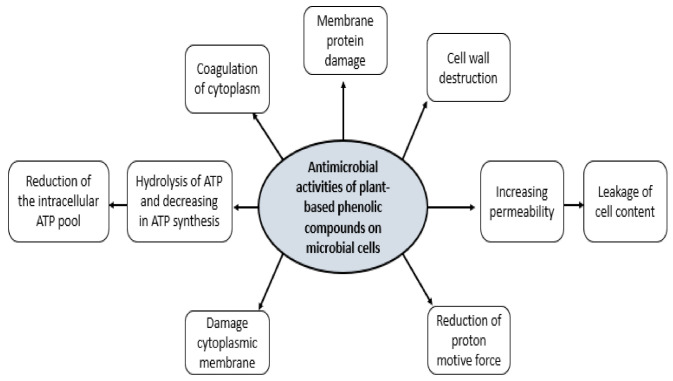
Antimicrobial activities of plant-based phenolic compound on microbial cells.

The antimicrobial mechanism of polyphenolic rich plant extracts includes the following [40]: (1) membrane disrupting molecules such as OH-group causes leakage of cellular content or interruption of the metabolic enzymes or dissipation of the cellular energy in its ATP form; (2) direct pH drop of the growth medium because of the increase in proton concentration, decrease of the internal cellular pH by dissociation of acid molecules and alteration of the cell membrane permeability; (3) the presence of an organic acid in the plant extract may impact on the NADH oxidation, thus removing the supply of the reducing agent to the electron transport system. Nevertheless, the exact molecular mechanism of the antimicrobial activity is yet to be well understood because plants and plant extracts contain a diverse range of bioactive molecules that are different in their configuration and chemical structure and that regularly function in synergy/antagonism with each other. This means, there may be several possible antimicrobial molecular mechanisms of action to impact the cell in many ways as shown in Figure 3.

The OH-group of a phenolic molecule is central to the antimicrobial activity of plant extracts. The OH-group can interact with the cell membrane of microorganism by hydrogen bonding [41]. This causes either the disruption of the membrane structure resulting in loss of cellular content, or the delocalization of electrons inducing the depolarization that impacts on the proton motive force, which decreases the pH gradient across the membrane and the level of the ATP pool, and ultimately leads to cell death. The number of OH-groups on the phenolic ring influences the intensity and severity of the antimicrobial activity. For example, Stojkovic and co-workers [42] studied the antimicrobial impact of caffeic acid, *p-*coumaric acid and rutin in food systems and found that caffeic acid had higher antimicrobial activity compared to *p-*coumaric acid because of the additional OH-group in the phenolic ring of caffeic acid.

The position of the OH-group(s) on the aromatic ring of the phenolic molecule also influences the antimicrobial effectiveness of plant-based preservatives [43]. For example, the difference in antimicrobial activity between thymol and carvacrol when tested against bacteria was attributed to the OH-group located at the meta position in thymol compared to the ortho position in carvacrol [44]. In another study, the antibacterial activity of flavonoids against Methicillin-resistant *Staphylococcus* aureus strains found that the OH-groups in positions 2’ of chalcones and position 5’ of flavanones and flavones increase their antimicrobial activity [45]. The position of the OH-group on the aromatic ring of polyphenols is, indeed, relevant in the delocalization of electron(s) from the cytoplasmic membrane which exerts an antimicrobial activity on bacteria [46].

## 6. Application of Polyphenolic-Rich Plants in Meat System

Plants including herbs and spices have been used for thousands of years in different applications including foods and beverages [47]. There are numerous trials conducted on the antioxidant and antimicrobial properties of a variety of plants used in comminuted meat as natural preservatives to inhibit microbial spoilage and to slow down the oxidative reactions. Only a few of the major plant species used will be discussed further to highlight their relevance in the comminuted meat processes.

### 6.1. Plant-Based Antioxidants Applied for Preservation in Meat Systems

Antioxidant activity is widely observed in a variety of plants and different parts of the plant including fruits, herbs, seeds, peels, leaves, rhizomes, and flowers. This activity is attributed to the presence of bioactive compounds. Several peer-reviewed publications reported the application of plants and plant extracts with success in preventing or slowing down the oxidative reactions in comminuted meats.

#### 6.1.1. Drumstick Leaves (*Moringa oleifera*)

*M. oleifera* is a native plant to sub-Himalayan tracts of India, Pakistan, Bangladesh, and Afghanistan. Its leaves contain important bioactive molecules including glucosinolates, isothiocyanates, zeatin, quercetin, betasitosterol caffeoylquinic acid, and kaempferol that all display antioxidant properties [48]. In goat patties, 0.1% of Moringa leaf extract was incorporated to investigate its impact in terms of oxidative stability [49]. The experiment was carried out in refrigeration condition over 15 days. The *M. oleifera* leaf extract was more effective than BHT in maintaining low thiobarbituric acid reactive substances (TBARS) numbers in precooked, chilled goat meat patties. Additionally, the sensory attributes of the product were not affected. In another study, the effect of *M. oleifera* leaf powder on the ferric antioxidant power (FRAP) and lipid oxidation during processing and storage of dry pork sausage was investigated [18]. The inclusion of *M. oleifera* leaf powder in the recipe was 0.5, 1 and 2 % of Moringa leaf powder—results indicated that the FRAP was increased and the level of TBARS was significantly lower compared to the control.

#### 6.1.2. Amla (*Emblica officinalis*)

Amla is a plant widely domesticated in subtropical areas of China, Indonesia, and Malaysia. Its fruits and seeds powder are used as ingredients in many foods for promotion of healthiness and longevity (Table 1) [50]; their main bioactive compounds include gallic acid, ellagic acid, quercetin, and kaempferol. Bariya and co-workers [51] investigated the antioxidant potential of Amla fruit and seed coat in goat patties. A basal recipe of minced goat meat was prepared and then mixed, separately, with Alma fruit and seed coat extracts. Goat patties incorporated with Amla fruit extract and Amla seed coat extract had lower TBARS values and free fatty acid values compared to the control. The authors recommended that goat meat patties containing Amla extracts from fruit and seed can last up to 21 days under vacuum packed refrigerated conditions without loss in quality. Similar results also showed that the addition of Amla extracts into a raw chicken meat batter will prevent lipid oxidation without compromising sensory attributes [52].

#### 6.1.3. Mountain Savory (*Saturaja montana* L.)

Mountain savory is a perennial semi-shrub that inhabits arid, sunny, and rocky terrains in the Mediterranean region, although this plant has also been domesticated throughout Europe. The main bioactive molecules with antioxidant properties are carvacrol, borneol, linalool, thymol, γ-terpinene, and ρ-cymene [71]. The effects of mountain savory as a natural antioxidant in mortadella-type sausages were investigated [72]. Batches of mortadella-type sausages were formulated with different concentrations of sodium nitrite (0, 100, and 200 mg/kg) and mountain savory essential oil at concentrations of 7.8, 15.6, and 31.25 µL/g. The mountain savory extract significantly reduced the oxidative reactions of the lipids in the mortadella-type sausages. However, the use of savory extract in high concentrations combined with a high level of sodium nitrite induced unfavorable sensory change in terms of color of the finished product.

#### 6.1.4. Green Tea (*Camellia sinensis*)

Green tea is an ever-green plant that grows in tropical and temperate regions. Green tea extract contains several polyphenolic molecules with antioxidant properties in which the predominant active compound is catechins [35]. Numerous studies have investigated the antioxidant properties of green tea in meat and meat products. For example, the effect of green tea as a natural antioxidant in uncured pork sausage showed that 0.05% of green tea in combination with 0.030% of black tea suppressed the TBARS value more efficiently than 0.010% of BHT without changing the organoleptic characteristics such as color, order, taste, texture, and overall acceptability [73]. In fact, the utilization of green tea extract as a natural antioxidant in Turkish dry-fermented sausage showed that green tea extract was more effective than BHT in reducing TBARS without affecting the sensory attribute of the meat product [74].

#### 6.1.5. Clove Extract (*Syzigium aromaticum* L.)

Cloves, flower buds of *Syzigium aromaticum* L., is a valuable plant originally domesticated in China. The main bioactive compound in cloves with antioxidant properties are eugenol and caryophyllene [75]. The effect of natural antioxidants including clove extract in beef patties where the basic formulation of patties included 90.8% beef lean meat, 8% beef back fat, and 1.2% salt with clove extract included at 0.1% concentration showed retardation in the increase in TBARS, demonstrating the ability of cloves to inhibit the oxidation of lipids in beef patties during cold storage [15]. Additionally, the sensory panel found that the beef patties with clove extract had better flavor and overall acceptability. A study on the effect of clove extract as a natural antioxidant in Chinese-style sausages where the clove extract was included in the recipe at concentrations of 0, 0.25%, 0.5%, 1%, and 2%; indicated that the 2% inclusion of clove extract had the highest inhibition of lipid oxidation [75]. This might be due to the high concentration of polyphenols, including eugenol and caryophyllene in the clove extract.

#### 6.1.6. Grape Seed Extract (*Vitis vinifera*)

Grape seeds are the by-products of the winery and grape juice industry. These seeds contain 5 to 8% of polyphenols cinnamic acids, benzoic acids, catechin, epicatechin, and gallic acid [16,76]. The effect of grape seed extract (GSE) as antioxidant in raw fresh minced beef maintained under a refrigerated condition of 4 °C for 10 days was investigated [77]. The basal formulation was fresh mince with (1) 50 mg GSE/kg of meat (2) 200 mg GSE/kg of meat (3) 1000 GSE/kg of meat, (4) negative control (without antioxidant), and positive control (with 0.01% of BHT/kg of meat). The TBARS were significantly reduced in all treated minced beef meat samples during storage compared to both negative and positive control. In line with these results, the inclusion of GSE added at 0.02% and 0.04% in a green beef sausage recipe extended the shelf life without affecting the sensory attributes [78].

### 6.2. Plant-Based Antimicrobial Applied for Preservation in Meat Systems

#### 6.2.1. Roselle Calyx Extract (*Hibiscus sabdariffa*)

Roselle calyx is a plant that grows in the tropic and subtropical regions. This plant is native to Asia and is now cultivated throughout the world and is known by different names such as roselle, hibiscus, jacaranda, red sorrel or karkade. The major bioactive compounds found in the roselle flowers are gallic acid, catechin, epicatechin, chlorogenic acid, protocatechuic acid and hydroxycinnamic acids [79]. The antimicrobial properties of *H. sabdariffa* extract to improve the shelf life of beef meat was investigated; slices of 10 g of beef meat were sprayed, under refrigeration conditions with 250, 500, 750, 1000, or 1250 mg/l of the hibiscus extracts and then stored for 10 days until microbial analysis [80]. Microorganisms that were tested in this study include *Escherichia coli*, *Salmonella enterica serovar T.*, *Staphylococcus aureus*, *Listeria monocytogenes* and *Bacillus cereus*. The hibiscus calyx extract showed an increase in the shelf life of the meat. In a similar study, the extracts of *H. sabdariffa* were mixed at 5 or 10 mg with ground beef under refrigeration conditions [81]. Food spoilage bacteria examined included *Salmonella typhimurium* DT104, *Escherichia coli* O157:H7, *L. monocytogenes*, *S. aureus*, and *B. cereus*; the Roselle calyx extract showed significant antimicrobial effects.

#### 6.2.2. Chestnut Inner Shell (*Castanea crenata* Mill)

*Castanea crenata* is a woody plant native to South Korea and Japan. Chestnut shell is estimated to represent about 10% of the weight of the chestnut, and it is removed during the peeling process [82]. This chestnut by-product contains a significant concentration of phenols and hydrolysable tannins [83]. The chestnut inner shell extracts (CISE) have been reported to have an antimicrobial effect in meat products. For example, the antimicrobial effect of CISE against *Campylobacter jejuni* in chicken meat was studied [84] The chicken meat was cut into small portions and mixed with CISE made up of different concentrations (0, 0.5, 1, and 2 mg/g of CISE dissolved in 90 mL of 1% peptone solution) and kept under refrigeration conditions (4 °C) or at 42 °C. CISE inhibited the growth of *C. jejuni* in chicken meat both at 4 °C and 42 °C—the authors recommended that this plant extract can be used as a natural antimicrobial in meat and meat products.

#### 6.2.3. *Acacia nilotica* Extract

*Acacia* is a genus in the Leguminosae family comprising 1350 species distributed in the warm and arid regions around the world [85,86]. The leaves, bark, and pods of *Acacia nilotica* has been reported to have antimicrobial activities because of the presence of bioactive molecules including catechin(s) and gallocatechin [87]. The antimicrobial properties of *A. nilotica* seed extracts (ANSE) were evaluated in chicken patties [62]. Formulation of chicken patties were performed by mixing minced chicken with various Acacia seed extract concentrations (0, 50, 100, and 150 mg/100mL distilled water) and storing the mince under refrigeration conditions (4 °C). The microorganisms that were tested include *E. coli* ATCC 10536, *S. typhimurium* ATCC 14028, *Y. enterocolitica* ATCC 27729, *Klebsiella pneumonia* ATCC 10031, *B. cereus* ATCC 14579 and *S. aureus* ATCC 29737 (Table 2). The shelf life of chicken patties was prolonged for 15 days under concentration of 150 mg ANSE/100 mL distilled water. In line with these findings, *A. nilotica* extract when used in beef patties at concentration of 1% and 2% in the beef burger recipe also showed strong antimicrobial effects [88].

#### 6.2.4. Carob Fruits (*Ceratonia Siliqua*)

*Ceratonia siliqua*, commonly known as Carob fruit, is considered an underutilized plant, native to Mediterranean regions including Northern Africa, Southern Europe, and the larger Mediterranean islands [13]. This plant has antimicrobial properties, and the major bioactive compounds include hydroxybenzoic acids, flavonols such as quercetin, myricetin, kaempferol and their glycosidic derivatives [97]. The antimicrobial activity of *C. siliqua* against Listeria in minced meat was evaluated [98]. Half of 25 g minced beef meat was inoculated with 2.10^2^ cfu/g of *L. monocytogens*/g of meat and thoroughly mixed for 3 min at room temperature. The extract of *Ceratonia siliqua* pods was then added to the 25 g inoculated minced mixture at concentrations of 0.1, 0.2, or 0.4 mg of *C. siliqua* extract/g of meat. The samples were stored under refrigeration conditions (7 °C) for 10 days. The *C. siliqua* extract inhibited the food pathogen, suggesting that this plant is a potential source of active ingredients suitable for the food and pharmaceutical industries.

#### 6.2.5. Lemongrass (*Cymbopogon citratus*)

Lemongrass (*C. citratus*) is a perennial and tall grass encompassing around 55 species and is domesticated in tropical and subtropical regions. The major antimicrobial compounds in Lemon grass include citral (65–86%), a mixture of terpenoids citral B (neral) and citral A (geraniol) [99]. The antimicrobial effects of Lemongrass oil on the quality characteristic of camel burgers stored under refrigeration conditions (4 °C) for 12 days was investigated [100]. The concentration of lemongrass in the green mix were 0.5%, 0.75%, and 1%. The camel burger formulated with 1% lemongrass showed the lowest total viable count and, in addition, recorded the highest in organoleptic characteristic assessment. Similarly, lemongrass (concentration of 1.0%) inhibited the growth of mesophilic and psychotrophic bacteria in fresh chicken sausage [101].

## 7. Limitations of Natural Preservatives when Applied in Food Systems

Several scientific studies acknowledge the antimicrobial and antioxidant properties of plant polyphenols. However, most of these studies have been performed in-vitro (not all have been reported in this review) and their application in meat systems (in-situ) might have different outcomes. In in-situ applications, the amount of these phenolic-rich plant preservatives required to have an effect, are frequently so high that these levels are not always organoleptically acceptable, whereas in the in-vitro applications, much lower amounts of the same plant extracts are needed to display the antimicrobial and antioxidant properties [102]. The probable explanation of the difference observed between the in-vitro and in-situ application of (poly)phenolic-rich plant extracts maybe because of the presence of other components in meat systems such as fat, proteins, carbohydrates, and salt which may have an impact on their bioactivity. Two aspects are crucial for the practicability of using natural preservatives in food systems [103]: (1) the change of sensory attributes of food such as color, smell, and texture when the natural preservative is added, and (2) the interactions of the natural preservative with other food ingredients in the system. Meats and meat products are commonly rejected by consumers when the natural preservative is in the form of plant essential oil, which is volatile and impart a strong flavor [104].

Furthermore, bioactive compounds’ concentration in herbs/spices may differ, for the same spices, depending on the extraction techniques, harvesting season and between geographical regions [86]. In addition, the bioactive compounds in the plant part (seed, leaf, root bark and stem bark) may not be the same in terms of quality and quantity [105]. For example, the main barrier encountered in the use of plant extracts, particularly essential oils, is the inability of the reproducibility of their activity [41]. This is because, although they consist of a diverse collection of chemical compounds, they have different qualitative and quantitative fluctuations in the concentration of the active molecule which influence their bioactivity. In addition, the cost, selectivity and safety of these extract should be considered as these could be limiting factors in the selection of solvent and technique of extraction [25]. Another aspect that warrants more scrutiny is the fact that many of these plant extracts are being marketed on their image of being “green”, yet some of the extraction methodologies such as solvent extraction is in fact aggressive and dangerous.

As mentioned, the amount of extract required to positively influence the shelf-life stability of the comminuted meat products, are frequently high and as a result, could influence other organoleptic attributes. Additionally, a number of papers published on plant-based preservatives do not in fact indicate the concentrations at which the extracts, and more importantly, the active components therein, are added to the meat, thus making it challenging to evaluate the efficacy thereof under in-situ conditions.

The legislative aspects of natural preservatives may pose problems in some instances. For example, garlic appears to be generally safe based on substantial tract records of consumption and use in foods including meats and meat products. However, there have been a number of scientific publications that established inconveniences, such as allergic reactions and alteration of platelet function and coagulation, that this polyphenolic-rich plant may cause [106]. Furthermore, the Food and Drug Administration (FDA) of the Unites States of America classifies estragole as a flavoring with as GRAS status whereas the EU prohibit it on the ground that it may be genotoxic. Clearly, discordances of regulations on natural food preservatives between regulatory bodies may be an additional impediment to their use in meat and meat products.

## 8. Regulations of Natural Food Preservatives

The most influential governing bodies that legislate, enforce the law, and oversee the approval and regulation of food additives in the World include the European Food Safety Authority (EFSA) in the European Union and the FDA. Another important organization involved in the risk assessment and studies on food preservatives and who regularly issue statements regarding food preservatives is the joint FAO/World Health Organization (WHO) Expert Committee on Food Additives, and the Codex Alimentarius [107].

The addition of natural preservatives in the food legislation and their subsequent approval for commercial use in the food industry require a comprehensive science-based evidences, full description of the product, manufacturing and storage methods, the safety report of the product, compliance with the Good Manufacturing Practice (GMP), ingredient warning, pack labelling and notification [108]. In the European Union (EU), regulation EC1333/2008 on food additives put in place a list of approved food additives that was published in full in regulation EU 1129/2011 [109]. According to this regulation, natural preservatives currently approved as food additives in the EU include rosemary, tocopherol-rich extracts (E 306), γ-tocopherol (E 308), δ-tocopherol (E 309), carotenes (E 160a), and annatto, bixin and norbixin (E 160b). However, considering meat, specific natural preservatives are approved as food additives. These include rosemary which is to be used only in dried sausage at maximum level of 100 mg/Kg or 150 mg/Kg in dehydrated meat only and Carotene to be used in sausage, pâtés and terrines at a maximum level of 20 mg/kg [110].

In the United States, the FDA regulates the dose and type of additives and preservatives allowed in meat and meat products. Natural preservatives with GRAS status are added in the Food Additive Status list issued by the U.S. Department of Agriculture (USDA). In the most recent ruling for GRAS preservatives published [111], all future GRAS reviews would be “self-determinations” of GRAS status by the notifier and encourages the manufacturer to provide a notification of the GRAS conclusion on the additive used. This recent ruling for GRAS preservatives has been misinterpreted and is allowing companies to make their own safety determination on GRAS substances they use without notifying the FDA [112]. There are two ways a substance can be classified as GRAS, based on: (1) “scientific procedure” that include human, animal, analytical and scientific studies to establish the safety of a substance. (2) “common use in food” defined as substantial history of consumption of a substance for use in food [113]. However, natural preservatives such as estragole is classified by FDA as a GRAS substance but prohibited as flavoring in the EU. On the other hand, Codex Alimentarius proposed a maximum carotenoid level of 20 mg/kg in fresh meat, poultry, and game (comminuted), but the United States of America (USA) does not adopt this proposal in its legislation.

In Australia and New Zealand, the Food Standards Australia New Zealand (FSANZ) is a bi-national self-governing statutory authority that put in place the food standard code and plays an important role in ensuring public health, providing suitable information about food including food additives. “Natural” claims on food products are particularly convincing to many Australians. However, there are no enforceable food standard regulating “natural additive” or the use of the term “natural”. This leaves consumers with only the uncertain protection of the Trade Practice Act of 1974 which forbids food manufacturers from misleading or deceiving consumers [12].

## 9. Conclusions

Microbial spoilage and oxidative reactions are the major problems that decrease the shelf-life of meat and meat products. The food industry extensively uses synthetic food preservatives to maintain the safety and quality of their products, but due to the potential health concerns these chemical compounds can cause, consumers are nowadays demanding for much healthier alternatives. In the food manufacturer communities, the interest of finding natural preservative to meet consumers requirement is trending. Polyphenolic-rich plants used as food preservatives offer the best alternative for the replacement of their synthetic counterparts. The common feature of these phenolic compounds is that they have one or more aromatic rings with one or more -OH group which are essential for their antimicrobial and antioxidant properties. Several studies on the application of polyphenolic-rich plants in comminuted meat reported improvement of the safety and quality of the finished products. However, the molecular mechanisms of polyphenol bioactivities are yet to be well understood. Furthermore, the amount of polyphenolic-rich plant required in the meat system for effective antimicrobial and antioxidant activities are often so high that these levels are not always organoleptically acceptable. Thus, studies on evaluating synergistic effects and optimization involving plant-based natural preservative to advance the knowledge in manufacturing meat products with longer shelf life and free from synthetic preservatives without compromising sensory attributes. On the aspect of the regulation of natural food preservatives, differences exist between regulatory bodies such as FDA and EU. Hence, harmonization of concepts, definitions, and approval requirements of food additives as well as food natural preservatives is required so that they are clearer and can benefit to the food manufacturers, academic institutions, and anyone else with an interest in food additives.

## Figures and Tables

**Table 1:** Bioactive rich plants as antioxidants preservatives used in comminuted meats.

Plant Sources	Scientific Name	Major Component	Part Used	Delivery System	Amount Added in Meat (%)	Meat System	Effect	References
Amla	*Emblica officinalis*	Gallic acid, ellagic acid, quercetin, and kaempferol.	Fruit, seed	Extract	-	Goat patties	Inhibition of lipid oxidation	[51]
Apple	*Malus domestica*	Lycopene	Peel and seed	Pomace powder	1-5	Indian traditional meat	Shelf-life extension	[53]
Black cumin	*Nigella sativa* L.	Thymoquinone, thymohydroquinone, dithymoquinone, p-cymene, and trans-anethol	Seed	Extract	1.5	Fresh minced beef	Lower level of TBARS	[54]
Black currant	*Ribes nigrum*	Anthocyanins	Fruits	Extract	>0.5	Pork patties	Inhibited lipid oxidation	[55]
Black pepper	*Piper nigrum*	Capsanthin and capsorubin	Fruit	Powder	1	Fresh pork sausage	Inhibition of lipid oxidation	[56]
Broccoli	*Brassica oleracea*	Phenolic acids and flavonoids	Leaf	extract	>0.1	Ground beef patties	Lower level of TBARS	[57]
Chilli pepper	*Capsicum annuum*	Capsaicin and dihydrocapsaicin	Fruit	Powder	2	Fresh pork sausage	Inhibition of lipid oxidation	[56]
Clove Extracts	*Syzigium aromaticum* L.	Eugenol and eugenyl acetate	Flower buds	Extract	0.1	Beef patties	Inhibition of lipid oxidation	[15]
Curry	*Murraya koenigii* L.	Tannic, gallic, caffeic, cinnamic, chlorogenic, ferulic, and vanillic acids	Leaf	Extract	2.5	Meat homogenate	Lower level of TBARS	[58]
drumstick	*M. oleifera*	Glucosinalates, isothiocyanates, zeatin, quercetin, betasitosterol caffeoylquinic and kaempferol	Leaf	Extract	0.1	Beef patty	Lower level of TBARS	[48]
Fatsia	*Aralia elata Seem*	Phenolic acid, Flavonoid, aromatic compounds	Leaf	Extract	1 and 5	Beef patties	Lower level of TBARS	[57]
Fenugreek	*Trigonella foenum-graecum*	Galactomannans, nicotinic acid, alkaloids, flavonoids	Seed	Powder	-	Rabbit sausage	Reduced lipid oxidation	[59]
Ginger	*Zingiber officinale* L.	Gingerol and shogaols	Rhizome	Peeled and shredded	1.5	Minced meat	Lower level of TBARS	[60]
Ginkgo biloba	-	Polyphenols	Leaf	Extract	0.05	Meatballs	Lower level of TBARS	[61]
Grape seed	*Vitis vinifera*	Catechin, epicatechin, gallic acid and phenolic acid	Seed	Extract	0.01	Fish mince	Lower level of TBARS	[16]
Green tea	*Camellia sinensis*	Catechin	Leaf	Extract	0.01	Fish mince	Lower level of TBARS	[16]
Hardy garden mum	*Chrysanthemum morifolium*	Polyphenolic compounds	Flower	Extract	1 and 2	Goat meat patties	Inhibit lipid and protein oxidation	[62]
Lotus	*Nelumbo nucifera*	Tartaric acid, citric acid, malic acid, succinic acid, tannin	Leaf	Leaf powder	6	Chicken patties	Lower level of TBARS	[63]
Lychee seed	*Litchi chinensis*	Epicatechin, procyanidin B4 and procyanidin B2	Fruit	Pericarp extract	>0.1	Sheep meat nugget	Lower level of TBARS	[64]
Mulberry	*Morus alba* L.	polyphenols, flavonoids and antho- cyanins Polyphenols, flavonoids, and anthocyanins	Leaf	Extract	>0.01	Ground beef	Lower level of TBARS	[65]
Myrtle	*Myrtus communis*	α-pinene, 1,8-cineole, limonene, linalool, α-terpineol	Leaf	Extract	-	Beef patties	Lower level of TBARS	[66]
Pomegranate	*Punica granatum*	Tannins, anthocyanins, and flavonoids	Fruit	Extracts	0.02	Ground pork meat	Lower level of TBARS	[67]
Rosemary	*Rosemarinus officinalis* L.	Carnosic acid, Caffeic acid, and rosmarinic acid	Leaves	Extract	>0.012	Pork meat	Inhibition of lipid oxidation	[68]
Turmeric	*curcuma longa*	Curcumin	Rhizome	Powder	3.5	Rabbit patties	Inhibition of lipid oxidation	[69]
Wattles	*Acacia nilotica*	Flavone, alkaloids, and gums	Seed	Extract	>0.1	Chicken patties	Shelf-life extension	[70]

**Table 2:** Some studies regarding the application of phenolic-rich extracts to comminuted meat products to enhance meat preservation.

Plant	Scientific Name	Meats	Amount Added in meat (%)	Delivery System	Targeted Species	Outcome	References
*Acacia nilotica*	*Acacia nilotica*	Minced chicken	-	Seed extract	*E. coli*, *S. typhimurium*, *Y. enterocolitica*, *K. pneumonia*, *B. cereus*, *S. aureus*	Growth inhibition	[70]
Bottlebrushes	*Callistemon citrinus*	Burgers	2	Leaves and flowers extracts	*L. monocytogens*	Growth inhibition	[89]
Chestnut	*Castanea sativa*	Chorizo	0.1	Leaves extracts	Total Viable counts	Microbial growth inhibition	[90]
Cranberry	*Vaccinium subg. Oxycoccus*	Cured Frankfurters	3	Powder	*Listeria monocytogens*	Growth inhibition	[91]
Drumsticks	*M. oleifera*	Poultry sausage	0.5	Ground powder	*E. coli*, *S.aureus*, Total plate counts	Growth inhibition	[92]
Grape seed	*Vitis vinifera*	Turkey Frankfurters	0.5	Seed extract	*E. coli* O157:H7, *L. monocytogenes*, *S. typhimurium*,	Contributed to the microbial reduction	[93]
Green tea	*Camellia sinensis*	Pork meatballs	0.03	Extract	*Mold*, *Yeast*, *Coliforms*	Growth inhibition	[94]
Lentisk	*Pistacia lentiscus* L.	Pork sausage	0.03	Extracts	Total Viable counts	Slowing microbial growth	[95]
Mustard	*Brassica juncea*	Bologna	5	Extract	*L. monocytogens*	Growth inhibition	[96]

## References

[1] FAO (2010). The State of Food Insecurity in the World Addressing Food Insecurity in Protracted Crises.

[2] Gonelimali F.D., Lin J., Miao W., Xuan J., Charles F., Chen M., Hatab S.R. (2018). Antimicrobial Properties and Mechanism of Action of Some Plant Extracts Against Food Pathogens and Spoilage Microorganisms. Front. Microbiol..

[3] Godfray H.C.J., Aveyard P., Garnett T., Hall J.W., Key T.J., Lorimer J., Pierrehumbert R.T., Scarborough P., Springmann M., Jebb S.A. (2018). Meat consumption, health, and the environment. Science.

[4] Berton-Carabin C.C., Ropers M.-H., Genot C. (2014). Lipid Oxidation in Oil-in-Water Emulsions: Involvement of the Interfacial Layer. Compr. Rev. Food Sci. Food Saf..

[5] D’Amore T., Di Taranto A., Berardi G., Vita V., Marchesani G., Chiaravalle A.E., Iammarino M. (2020). Sulfites in meat: Occurrence, activity, toxicity, regulation, and detection. A comprehensive review. Compr. Rev. Food Sci. Food Saf..

[6] Binstok G.C.C., Varelab O., Gerschenson L.N. (1998). Sorbate ± nitrite reactions in meat products. Food Res. Int..

[7] Hoang Y.T.H., Vu A.T.L. (2016). Sodium Benzoate and Potassium Sorbate in Processed Meat Products Collected in Ho Chi Minh City, Vietnam. Int. J. Adv. Sci. Technol..

[8] Yehye W.A., Rahman N.A., Ariffin A., Abd Hamid S.B., Alhadi A.A., Kadir F.A., Yaeghoobi M. (2015). Understanding the chemistry behind the antioxidant activities of butylated hydroxytoluene (BHT): A review. Eur. J. Med. Chem..

[9] Rajendran C., Mahesh S., Jayathilakan K. (2019). Advances in Meat Preservation and Safety. Int. J. Sci. Res..

[10] Osterbauer K.J., King A.M., Seman D.L., Milkowski A.L., Glass K.A., Sindelar J.J. (2017). Effects of Nitrite and Erythorbate on *Clostridium perfringens* Growth during Extended Cooling of Cured Ham. J. Food Prot..

[11] Aziz M., Karboune S. (2018). Natural antimicrobial/antioxidant agents in meat and poultry products as well as fruits and vegetables: A review. Crit. Rev. Food Sci. Nutr..

[12] Williams P.G., Markoska J., Chachay V., Anne M. ‘Natural’ Claims on Foods: A Review of Regulations and a Pilot Study of the Views of Australian Consumers 2009. https://ro.uow.edu.au/hbspapers/121.

[13] Ahmad S.R., Gokulakrishnan P., Giriprasad R., Yatoo M.A. (2015). Fruit-based Natural Antioxidants in Meat and Meat Products: A Review. Crit. Rev. Food Sci. Nutr..

[14] Hęś M., Gramza-Michałowska A. (2017). Effect of Plant Extracts on Lipid Oxidation and Changes in Nutritive Value of Protein in Frozen-Stored Meat Products. J. Food Process. Preserv..

[15] Zahid M.A., Choi J.Y., Seo J.K., Parvin R., Ko J., Yang H.S. (2020). Effects of clove extract on oxidative stability and sensory attributes in cooked beef patties at refrigerated storage. Meat Sci..

[16] Ozen B.O., Soyer A. (2018). Effect of plant extracts on lipid and protein oxidation of mackerel (*Scomber scombrus*) mince during frozen storage. J. Food Sci. Technol..

[17] Zhang Y., Henning S.M., Lee R.P., Huang J., Zerlin A., Li Z., Heber D. (2015). Turmeric and black pepper spices decrease lipid peroxidation in meat patties during cooking. Int. J. Food Sci. Nutr..

[18] Mukumbo F.E., Descalzo A.M., Collignan A., Hoffman L.C., Servent A., Muchenje V., Arnaud E. (2019). Effect of *Moringa oleifera* leaf powder on drying kinetics, physico-chemical properties, ferric reducing antioxidant power, α-tocopherol, β-carotene, and lipid oxidation of dry pork sausages during processing and storage. J. Food Process. Preserv..

[19] Das A.K., Nanda P.K., Madane P., Biswas S., Das A., Zhang W., Lorenzo J.M. (2020). A comprehensive review on antioxidant dietary fibre enriched meat-based functional foods. Trends Food Sci. Technol..

[20] Efenberger-Szmechtyk M., Nowak A., Czyzowska A. (2021). Plant extracts rich in polyphenols: Antibacterial agents and natural preservatives for meat and meat products. Crit. Rev. Food Sci. Nutr..

[21] Leopoldini M., Russo N., Toscano M. (2011). The molecular basis of working mechanism of natural polyphenolic antioxidants. Food Chem..

[22] Michalak A. (2006). Phenolic Compounds and Their Antioxidant Activity in Plants Growing under Heavy Metal Stress. Pol. J. Environ. Stud..

[23] Jiang J., Xiong Y.L. (2016). Natural antioxidants as food and feed additives to promote health benefits and quality of meat products: A review. Meat Sci..

[24] Brewer M.S. (2011). Natural Antioxidants: Sources, Compounds, Mechanisms of Action, and Potential Applications. Compr. Rev. Food Sci. Food Saf..

[25] Qing-Wen Z., Li-Gen L., Wen-Cai Y. (2018). Techniques for extraction and isolation of natural products: A comprehensive review. Chin. Med..

[26] Machado A., Pereira A.L.D., Barbero G.F., Martinez J. (2017). Recovery of anthocyanins from residues of *Rubus fruticosus*, *Vaccinium myrtillus* and *Eugenia brasiliensis* by ultrasound assisted extraction, pressurized liquid extraction and their combination. Food Chem..

[27] Vilkhu K., Mawson R., Simons L., Bates D. (2008). Applications and opportunities for ultrasound assisted extraction in the food industry—A review. Innov. Food Sci. Emerg. Technol..

[28] Hirondart M., Rombaut N., Fabiano-Tixier A.S., Bily A., Chemat F. (2020). Comparison between Pressurized Liquid Extraction and Conventional Soxhlet Extraction for Rosemary Antioxidants, Yield, Composition, and Environmental Footprint. Foods.

[29] Singh P.P., Saldaña M.D.A. (2011). Subcritical water extraction of phenolic compounds from potato peel. Food Res. Int..

[30] Wijngaard H., Hossain M.B., Rai D.K., Brunton N. (2012). Techniques to extract bioactive compounds from food by-products of plant origin. Food Res. Int..

[31] Azmir J., Zaidul I.S.M., Rahman M.M., Sharif K.M., Mohamed A., Sahena F., Jahurul M.H.A., Ghafoor K., Norulaini N.A.N., Omar A.K.M. (2013). Techniques for extraction of bioactive compounds from plant materials: A review. J. Food Eng..

[32] Tir R., Dutta P.C., Badjah-Hadj-Ahmed A.Y. (2012). Effect of the extraction solvent polarity on the sesame seeds oil composition. Eur. J. Lip. Sci. Technol..

[33] Salminen J.-P. (2003). Effects of sample drying and storage, and choice of extraction solvent and analysis method on the yield of birch leaf hydrolyzable tannins. J. Chem. Ecol..

[34] Anita S., Casimir C.A., Weiguang Y., Joan F., Gerard K. (2007). Effect of Storage Conditions on the Biological Activity of Phenolic Compounds of Blueberry Extract Packed in Glass Bottles. J. Agric. Food Chem..

[35] Senanayake S.P.J.N. (2013). Green tea extract: Chemistry, antioxidant properties and food applications—A review. J. Funct. Foods.

[36] Kelly E.H., Anthony R.T., Dennis J.B. (2002). Flavonoid antioxidants: Chemistry, metabolism and structure-activity relationships. J. Nutr. Biochem..

[37] Cao G., Emin S., Ronald L.P. (1997). Antioxidant And Prooxidant Behavior Of Flavonoids: Structure—Activity Relationships. Free Rad. Biol. Med..

[38] Saskia A.B.E.V.A., Marcel J.D.G., Michel N.J.T., Gabrielle D.D.K., van der Wim J.F.V., Aalt B. (1996). A Quantum Chemical Explanation of the Antioxidant Activity of Flavonoids. Chem. Res. Toxicol..

[39] BozÏidar L.M., Sonja M.D., Jasna M.C.A.-B. (1998). Antioxidative activity of phenolic compounds on the metal-ion breakdown of lipid peroxidation system. Food Chem..

[40] Rabin G., Saeed A.H., Salam I. (2015). Plant extracts as antimicrobials in food products. Handbook of Natural Antimicrobials for Food Safety and Quality.

[41] Chibane L.B., Degraeve P., Ferhout H., Bouajila J., Oulahal N. (2019). Plant antimicrobial polyphenols as potential natural food preservatives. J. Sci. Food Agric..

[42] Stojkovic D., Petrovic J., Sokovic M., Glamoclija J., Kukic-Markovic J., Petrovic S. (2013). In situ antioxidant and antimicrobial activities of naturally occurring caffeic acid, p-coumaric acid and rutin, using food systems. J. Sci. Food Agric..

[43] Dorman H.J.D., Dean S.G. (2000). Antimicrobial agents from plants: Antibacterial activity of plant volatile oils. J. Appl. Microbiol..

[44] Gyawali R., Ibrahim S.A. (2014). Natural products as antimicrobial agents. Food Control..

[45] Alcaraz L.E., Blanco S.E., Puig O.N., Tomas F., Ferretti F.H. (2000). Antibacterial activity of flavonoids against methicillin-resistant *Staphylococcus aureus* strains. J. Theor. Biol..

[46] Ultee A., Bennik M.H., Moezelaar R. (2002). The phenolic hydroxyl group of carvacrol is essential for action against the food-borne pathogen *Bacillus cereus*. Appl. Environ. Microbiol..

[47] Olatunde O.O., Benjakul S. (2018). Natural Preservatives for Extending the Shelf-Life of Seafood: A Revisit. Compr. Rev. Food Sci. Food Saf..

[48] Jed W.F., Amy T.Z., Paul T. (2001). The chemical diversity and distribution of glucosinolates and isothiocyanates among plants. Phytochemistry.

[49] Das A.K., Rajkumar V., Verma A.K., Swarup D. (2012). *Moringa oleiferia* leaves extract: A natural antioxidant for retarding lipid peroxidation in cooked goat meat patties. Int. J. Food Sci. Technol..

[50] Xiaoli L., Chun C., Mouming Z., Jinshui W., Wei L., Bao Y., Yueming J. (2008). Identification of phenolics in the fruit of emblica (*Phyllanthus emblica* L.) and their antioxidant activities. Food Chem..

[51] Bariya A.R., Patel A.S., Gamit V.V., Bhedi K.R., Parmar R.B. (2018). Assessment of Antioxidant and Sensory Properties of Amla (*Emblica officinalis*) Fruit and Seed Coat Powder Incorporated Cooked Goat Meat Patties. Int. J. Curr. Microbiol. Appl. Sci..

[52] Kumar Y., Langoo B.A. (2015). Effects of Aloe, Green Tea, and Amla Extracts on Microbiological and Oxidative Parameters of Refrigerated Raw Meat Batter. Agric. Res..

[53] Sajad A.R., Rehana A., Masoodi F.A., Adil G., Wani S.M. (2015). Utilization of apple pomace powder as a fat replacer in goshtaba: A traditional meat product of Jammu and Kashmir, India. J. Food Meas. Charact..

[54] Chauhan P., Das A.K., Nanda P.K., Kumbhar V., Yadav J.P. (2018). Effect of *Nigella sativa* seed extract on lipid and protein oxidation in raw ground pork during refrigerated storage. Nutr. Food Sci..

[55] Jia N., Kong B., Liu Q., Diao X., Xia X. (2012). Antioxidant activity of black currant (*Ribes nigrum* L.) extract and its inhibitory effect on lipid and protein oxidation of pork patties during chilled storage. Meat Sci..

[56] Luis M., Irene C., Jose A., Bel T., Pedro R. (2006). Effect of *Capsicum annuum* (Red Sweet and Cayenne) and *Piper nigrum* (Black and White) Pepper Powders on the Shelf Life of Fresh Pork Sausages Packaged in Modified Atmosphere. J. Food Sci..

[57] Kim S.-J., Cho A.R., Han J. (2013). Antioxidant and antimicrobial activities of leafy green vegetable extracts and their applications to meat product preservation. Food Control..

[58] Yogesh K., Jha S.N., Yadav D.N. (2012). Antioxidant Activities of *Murraya koenigii* (L.) Spreng Berry Extract: Application in Refrigerated (4 ± 1 °C) Stored Meat Homogenates. Agric. Res..

[59] Engy F.Z. (2018). Incorporation of Fenugreek Seed Powder In The Manufacturing of Rabbit Sausage and its effects on the Quality Properties During Storage. J. Adv. Food Sci. Technol..

[60] Draszanowska A., Karpinska-Tymoszczyk M., Olszewska M.A. (2020). The effect of ginger rhizome and refrigerated storage time on the quality of pasteurized canned meat. Food Sci. Technol. Int..

[61] Kobus-Cisowska J., Flaczyk E., Rudzinska M., Kmiecik D. (2014). Antioxidant properties of extracts from *Ginkgo biloba* leaves in meatballs. Meat Sci..

[62] Khan I.A., Xu W., Wang D., Yun A., Khan A., Zongshuai Z., Ijaz M.U., Yiqun C., Hussain M., Huang M. (2020). Antioxidant potential of *chrysanthemum morifolium* flower extract on lipid and protein oxidation in goat meat patties during refrigerated storage. J. Food Sci..

[63] Yun-Sang C., Ji-Hun C., Hack-Youn K., Hyun-Wook K., Mi-Ai L., Hai-Jung C., Sung K.L., Cheon-Jei K. (2011). Effect of Lotus (*Nelumbo nucifera*) Leaf Powder on the Quality Characteristics of Chicken Patties in Refrigerated Storage. Korean J. Food Sci. Anim..

[64] Arun K.D., Vincent R., Pramod K.N., Pranav C., Soubhagya R.P., Subhasish B. (2016). Antioxidant Efficacy of Litchi (*Litchi chinensis Sonn*.) Pericarp Extract in Sheep Meat Nuggets. Antioxidants (Basel).

[65] Xinzhuang Z., Deyong L., Qingxiang M., Cui H., Liping R. (2016). Effect of Melberry leaf Extract on Color, Lipid Oxidation, Antioxidanr Enzyme, Active and Oxidative bBreakdown Products of Raw ground Beef during Refrigerated Storage. J. Food Qual..

[66] Akarpat A., Turhan S., Ustun N.S. (2008). Effects of Hot Water Extracts From Myrtle, Rosemary, Nettle And Lemon Balm Leaves On Lipid Oxidation And Color Of Beef Patties During Frozen Storage. J. Food Process. Preserv..

[67] Qin Y.-Y., Zhang Z.-H., Li L., Xiong W., Shi J.-Y., Zhao T.-R., Fan J. (2013). Antioxidant effect of pomegranate rind powder extract, pomegranate juice, and pomegranate seed powder extract as antioxidants in raw ground pork meat. Food Sci. Biotechnol..

[68] Liu S., Zhang R., Fan L., Ma Y., Xiang Q. (2020). Effect of rosemary extract on lipid oxidation of cooked pork during simulated gastric digestion. J. Sci. Food Agric..

[69] Mancini S., Preziuso G., Paci G. (2016). Effect of turmeric powder (*Curcuma longa* L.) and ascorbic acid on antioxidant capacity and oxidative status in rabbit burgers after cooking. World Rabbit. Sci..

[70] Babiker E.E., Al-Juhaimi F.Y., Alqah H.A., Adisa A.R., Adiamo O.Q., Ahmed I.A.M., Alsawmahi O.N., Ghafoor K., Ozcan M.M. (2019). The effect of *Acacia nilotica* seed extract on the physicochemical, microbiological and oxidative stability of chicken patties. J. Food Sci. Technol..

[71] Hassanein H.D., Said A.A.H., Abdelmohsen M.M. (2014). Antioxidant polyphenolic constituents of *Satureja montana* L. Growing in Egypt. Int. J. Pharm. Sci..

[72] de Oliveira T.L.C., de Carvalho S.M., de Araújo Soares R., Andrade M.A., Cardoso M.D.G., Ramos E.M., Piccoli R.H. (2012). Antioxidant effects of *Satureja montana* L. essential oil on TBARS and color of mortadella-type sausages formulated with different levels of sodium nitrite. LWT-Food Sci. Technol..

[73] Jayawardana B.C., Viraji B.W., Thotawattage G.H., Dharmasena V.A.K.I., Liyanage R. (2019). Black and green tea (*Camellia sinensis* L.) extracts as natural antioxidants in uncured pork sausages. J. Food Process. Preserv..

[74] Bozkurt H. (2006). Utilization of natural antioxidants: Green tea extract and *Thymbra spicata* oil in Turkish dry-fermented sausage. Meat Sci..

[75] Huiyun Z., Xinyan P., Xinling L., Jingjuan W., Xinyu G. (2017). The Application of Clove Extract Protects Chinese-style Sausages against Oxidation and Quality Deterioration. Korean J. Food Sci. Anim. Resour..

[76] John S., Jianmel Y., Joseph E.P., Yukio K. (2003). Polyphenolics in Grape Seeds—Biochemistry and Functionality. J. Food Med..

[77] Reham A.A., Shimaa N.E. (2017). Grape Seed Extract as Natural Antioxidant and Antibacterial in Minced Beef. PSM Biol. Res..

[78] El-Zainy A.R., Morsy A.E., Sedki A.G., Mosa N.M. (2016). Polyphenols Grape seeds extract as antioxidant and antimicrobial in Beef sausage. Int. J. Curr. Sci..

[79] Purbowati I.S.M., Maksum A. (2019). The antioxidant activity of Roselle (*Hibiscus sabdariffa Linii*) phenolic compounds in different variations microwave-assisted extraction time and power. IOP Conf. Ser. Earth Envirion. Sci..

[80] Marquez-Rodriguez A.S., Nevarez-Baca S., Lerma-Hernandez J.C., Hernandez-Ochoa L.R., Nevarez-Moorillon G.V., Gutierrez-Mendez N., Munoz-Castellanos L.N., Salas E. (2020). In Vitro Antibacterial Activity of *Hibiscus sabdariffa* L. Phenolic Extract and Its In Situ Application on Shelf-Life of Beef Meat. Foods.

[81] Chao C.Y., Yin M.C. (2009). Antibacterial effects of *roselle calyx* extracts and protocatechuic acid in ground beef and apple juice. Foodborne Pathog. Dis..

[82] Echegaray N., Gómez B., Barba F.J., Franco D., Estévez M., Carballo J., Marszałek K., Lorenzo J.M. (2018). Chestnuts and by-products as source of natural antioxidants in meat and meat products: A review. Trends Food Sci. Technol..

[83] Ja-Young H., In-Kyeong H., Jae-Bok P. (2001). Analysis of Physicochemical Factors Related to the Automatic Pellicle Removal in Korean Chestnut (*Castanea crenata*). J. Agric. Food Chem..

[84] Lee N.-K., Jung B.S., Na D.S., Yu H.H., Kim J.-S., Paik H.-D. (2016). The impact of antimicrobial effect of chestnut inner shell extracts against *Campylobacter jejuni* in chicken meat. LWT-Food Sci.Technol..

[85] Maslin B.R., Miller J.T., Seigler D.S. (2003). Overview of the generic status of Acacia (Leguminosae: Mimosoideae). Aust. Syst. Bot..

[86] Adiamo O.Q., Netzel M.E., Hoffman L.C., Sultanbawa Y. (2019). Acacia seed proteins: Low or high quality? A comprehensive review. Compr. Rev. Food Sci. Food Saf..

[87] Sadiq M.B., Hanpithakpong W., Tarning J., Anal A.K. (2015). Screening of phytochemicals and in vitro evaluation of antibacterial and antioxidant activities of leaves, pods and bark extracts of *Acacia nilotica* (L.) Del. Ind. Crop. Prod..

[88] Al-Juhaimi F.Y., Almusallam I.A., Ahmed I.A.M., Ghafoor K., Babiker E.E. (2020). Potential of *Acacia nilotica* fruit flesh extract as an anti-oxidative and anti-microbial agent in beef burger. J. Food Process. Preserv..

[89] Fayemi P.O., Öztürk I., Özcan C., Muguruma M., Yetim H., Sakata R., Ahhmed A. (2017). Antimicrobial activity of extracts of *Callistemon citrinus* flowers and leaves against *Listeria monocytogenes* in beef burger. J. Food Meas. Charact..

[90] Lorenzo J.M., González-Rodríguez R.M., Sánchez M., Amado I.R., Franco D. (2013). Effects of natural (grape seed and chestnut extract) and synthetic antioxidants (buthylatedhydroxytoluene, BHT) on the physical, chemical, microbiological and sensory characteristics of dry cured sausage “chorizo”. Food Res. Int..

[91] Xi Y., Sullivan G.A., Jackson A.L., Zhou G.H., Sebranek J.G. (2012). Effects of natural antimicrobials on inhibition of *Listeria monocytogenes* and on chemical, physical and sensory attributes of naturally-cured frankfurters. Meat Sci..

[92] Jayawardana B.C., Liyanage R., Lalantha N., Iddamalgoda S., Weththasinghe P. (2015). Antioxidant and antimicrobial activity of drumstick (*Moringa oleifera*) leaves in herbal chicken sausages. LWT-Food Sci. Technol..

[93] Gadang V.P., Hettiarachchy N.S., Johnson M.G., Owens C. (2008). Evaluation of antibacterial activity of whey protein isolate coating incorporated with nisin, grape seed extract, malic acid, and EDTA on a Turkey frankfurter system. J. Food Sci..

[94] Price A., Diaz P., Banon S., Garrido M.D. (2013). Natural extracts versus sodium ascorbate to extend the shelf life of meat-based ready-to-eat meals. Food Sci. Technol. Int..

[95] George B., Antia O., Evgenia Y., Vassilis G., Vlasios G. (2015). Antioxidant and Antimicrobial Effects of *Pistacia lentiscus* L. Extracts in Pork Sausages. Food Technol. Biotech..

[96] Lara-Lledo M., Olaimat A., Holley R.A. (2012). Inhibition of *Listeria monocytogenes* on bologna sausages by an antimicrobial film containing mustard extract or sinigrin. Int. J. Food Microbiol..

[97] Goulas V., Stylos E., Chatziathanasiadou M.V., Mavromoustakos T., Tzakos A.G. (2016). Functional Components of Carob Fruit: Linking the Chemical and Biological Space. Int. J. Mol. Sci..

[98] Hsouna A.B., Trigui M., Mansour R.B., Jarraya R.M., Damak M., Jaoua S. (2011). Chemical composition, cytotoxicity effect and antimicrobial activity of *Ceratonia siliqua* essential oil with preservative effects against Listeria inoculated in minced beef meat. Int. J. Food Microbiol..

[99] Josea C., Critina T., Jaime R., Guillermo-Hirschmann G. (2005). Free Radical Scavengers and Antioxidants from Lemongrass (*Cymbopogon citratus* (DC.) Stapf.). J. Agric. Food Chem..

[100] Ceruso M., Clement J.A., Todd M.J., Zhang F., Huang Z., Anastasio A., Pepe T., Liu Y. (2020). The Inhibitory Effect of Plant Extracts on Growth of the Foodborne Pathogen, *Listeria monocytogenes*. Antibiotics (Basel).

[101] Boeira C.P., Piovesan N., Soquetta M.B., Flores D.C.B., Lucas B.N., Rosa C.S.D., Terra N.N. (2018). Extraction of bioactive compounds of lemongrass, antioxidant activity and evaluation of antimicrobial activity in fresh chicken sausage. Ciência Rural.

[102] Martínez-Graciá C., González-Bermúdez C.A., Cabellero-Valcárcel A.M., Santaella-Pascual M., Frontela-Saseta C. (2015). Use of herbs and spices for food preservation: Advantages and limitations. Curr. Opin. Food Sci..

[103] Devlieghere F., Vermeiren L., Debevere J. (2004). New preservation technologies: Possibilities and limitations. Int. Dairy J..

[104] Lourenco S.C., Moldao-Martins M., Alves V.D. (2019). Antioxidants of Natural Plant Origins: From Sources to Food Industry Applications. Molecules.

[105] Darfour B., Asare I.K., Ofosu D.O., Achel D.G., Achoribo E.S., Agbenyegah S. (2014). The Effect of Different Drying Methods on the Phytochemicals and Radical Scavenging Activity of Ceylon Cinnamon (*Cinnamomum zeylanicum*) Plant Parts. Eur. J. Med. Plants.

[106] Borreli R.A., Capasso R., Izzo A.A. (2007). Garlic *(Allium sativum* L.): Adverse effects and drug interactions in humans. Mol. Nutr. Food Res..

[107] Carocho M., Morales P., Ferreira I.C.F.R. (2018). Antioxidants: Reviewing the chemistry, food applications, legislation and role as preservatives. Trends Food Sci. Technol..

[108] Dreger M., Wielgus K. (2013). Application of essential oils as natural cosmetic preservatives. Herba Pol..

[109] EU (2008). European Parliament and the Council of the European Union Regulation (EC) No 1333/2008 of the European Parliament and of the Council of 16 December 2998 on food additives. Off. J. Eur. Union..

[110] Manessis G., Kalogianni A.I., Lazou T., Moschovas M., Bossis I., Gelasakis A.I. (2020). Plant-Derived Natural Antioxidants in Meat and Meat Products. Antioxidants (Basel).

[111] US Food and Drug Administration Substances Generally Recognized as Safe, Final Rule. Federal Register 81 (17 August 2016): 54960–55055. https://www.gpo.gov/fdsys/pkg/FR-2016-08-17/pdf/2016-19164.pdf.

[112] Maffini M.V., Neltner T.G., Vogel S. (2017). We are what we eat: Regulatory gaps in the United States that put our health at risk. PLoS Biol..

[113] Sinopoli D.A. (2013). What Exactly is a Food Additive? A Comparative Analysis between the European Union and the United States on the Scope and Function of Food Additives.

